# Teacher support and classroom silence in vocational higher education: a serial mediation analysis through speaking self-efficacy and anxiety

**DOI:** 10.3389/fpsyg.2026.1844412

**Published:** 2026-05-22

**Authors:** Yun Zhang, Tinglei Liu

**Affiliations:** 1 Hebei Petroleum University of Technology, Chengde, Hebei, China; 2 Hebei Normal University for Nationalities, Chengde, Hebei, China; 3 Faculty of Modern Language and Communication, Universiti Putra Malaysia, Serdang, Selangor, Malaysia

**Keywords:** classroom silence, serial mediation, speaking anxiety, speaking self-efficacy, teacher support, vocational education

## Abstract

**Background:**

Classroom silence is common among vocational undergraduates and may limit students**’** opportunities for interaction, feedback, and classroom participation. However, the psychological pathways linking teacher support to classroom silence remain insufficiently specified in this population. This cross-sectional study tested a theoretically specified serial mediation model in which speaking self-efficacy and speaking anxiety were examined as mechanisms connecting perceived teacher support with classroom silence among Chinese vocational undergraduates.

**Methods:**

This cross-sectional study was conducted among vocational undergraduates from eight universities in Hebei Province, China. Data on perceived teacher support, classroom speaking self-efficacy, speaking anxiety, and classroom silence tendency were assessed using self-report scales, and 1,533 valid responses were included after data-quality screening. Structural equation modeling and bias-corrected bootstrapping with 5,000 resamples were used to estimate direct and indirect statistical pathways among these variables.

**Results:**

Perceived teacher support did not show a significant direct association with classroom silence (*β* = 0.101, *p* = 0.495). Higher perceived teacher support was associated with lower classroom silence through higher speaking self-efficacy (*β* = **−**0.104, 95% CI [**−**0.132, **−**0.077]). In addition, the serial pathway through higher speaking self-efficacy and lower speaking anxiety was significant (*β* = **−**0.127, 95% CI [**−**0.151, **−**0.104]), and the total indirect effect was also significant (*β* = **−**0.166, 95% CI [**−**0.194, **−**0.139]). Mediation analysis further showed a small positive indirect effect through speaking anxiety alone (*β* = 0.066, 95% CI [0.043, 0.090]), whereas model fit indices indicated an acceptable fit (NFI = 0.958, SRMR = 0.046).

**Conclusion:**

Teacher support was associated with lower classroom silence primarily through higher speaking self-efficacy and lower speaking anxiety. Because the study was cross-sectional, the findings should be interpreted as theory-guided associations rather than evidence of causal effects. Interventions that strengthen communicative confidence and address speaking anxiety may help promote classroom participation among vocational undergraduates.

## 1 Introduction

China’s vocational higher education system occupies a distinctive position within the national educational landscape. It combines practical technical training with academic instruction and has increasingly been positioned as a major route for cultivating applied professionals rather than as a peripheral alternative to academic higher education (Xue and Li, 2022; Wu et al., 2024). The expansion of four-year vocational undergraduate institutions reflects national efforts to upgrade this sector and align graduate competencies with changing labor market demands (Chen et al., 2025). Recent policy initiatives, particularly the Outline of the Plan for Building China into an Education Powerhouse (2024–2035), have further emphasized communication ability alongside technical competence (Shi and Li, 2025). Yet classroom interaction in vocational higher education often remains constrained by persistent silence (Jia et al., 2022).

This context is important because vocational undergraduates often occupy an ambivalent social position. On the one hand, they are expected to acquire work-oriented expertise, participate in applied learning, and communicate effectively in classroom and workplace settings. On the other hand, vocational education in China has historically been associated with lower academic status and with students who did not enter more academically selective universities. Such institutional and social meanings may make classroom speaking psychologically consequential: participation can be interpreted not only as a learning behavior but also as a public test of competence, confidence, and face. For readers outside China, this background helps explain why classroom silence in vocational higher education cannot be reduced to simple passivity or lack of interest.

This “silent classroom” phenomenon extends beyond mere absence of verbal participation. Unlike reflective silence that facilitates deep processing and contemplation, the silence documented in vocational classrooms frequently manifests as defensive withdrawal from public discourse, driven by fear of negative evaluation and fragile academic self-concepts exacerbated by perceived academic failure (Looney, 2022; Yue et al., 2022). Many vocational students internalize the perception of having “failed” competitive academic pathways, particularly the Gaokao university entrance examination, resulting in learned helplessness that paradoxically coexists with explicit disavowal of silence as desirable behavior (Juma et al., 2022). This may create a reinforcing feedback loop wherein instructors, interpreting silence as apathy or ignorance, retreat into lecture-centric pedagogies that further marginalize student voices and reinforce passive learning roles (Cents-Boonstra et al., 2021; Hoferichter et al., 2022).

Addressing classroom silence is therefore an important educational and psychological issue. Recent evidence suggests that silence may be better understood as a regulated participation strategy rather than as simple disengagement. Psychological factors associated with bystander passivity, especially self-efficacy and anxiety, may also help explain why students remain silent in class (Yang and Gao, 2023; Beavon et al., 2024). Clarifying these mechanisms has practical value. A clearer account of why students remain silent may help educators design more targeted ways to support participation, particularly among vocational undergraduates, who remain underrepresented in higher education research (Han et al., 2023; Zhang and Qian, 2024). Teacher support may be one important contextual factor in this process. A supportive teacher can create a classroom climate that feels safer, more respectful, and more encouraging (Tao et al., 2022). However, support may not translate directly into speaking behavior. Its association with classroom silence may depend on how students interpret and internalize that support. In particular, teacher support may strengthen students’ confidence in their ability to speak in class (Jiang and Tanaka, 2022), while also reducing the anxiety associated with public participation. From this perspective, speaking self-efficacy and speaking anxiety may function as more proximal psychological mechanisms linking classroom context to silence behavior.

The present study therefore examines whether perceived teacher support is associated with classroom silence indirectly through speaking self-efficacy and speaking anxiety. We propose that teacher support is more likely to relate to lower silence when it is internalized as communicative competence and emotional security. Accordingly, teacher support is expected to show no substantial direct statistical pathway to classroom silence once self-efficacy and anxiety are taken into account. Instead, its association with silence is expected to operate through a serial mediation pathway.

The proposition that distal contextual support reaches its outcomes through more proximal psychological resources extends beyond classroom silence. A converging set of recent studies has documented this same internal architecture in adjacent educational settings: when leadership support is linked to teacher burnout, much of the signal is carried by organizational support and resilience (Karacabey et al., 2025); when leadership is linked to organizational change, instructional strategy self-efficacy and emerging anxiety constructs do similar mediating work (Atasoy and Özkul, 2026); and at the individual level, self-efficacy is associated with lower classroom management anxiety through self-directed learning among pre-service teachers (Özkul, 2025). What these literatures share with the present model is not their populations but their structural logic—contextual variables do not reach behavioural outcomes directly, but are converted, through psychological resources, into the affective states that ultimately differentiate engagement from withdrawal.

## 2 Materials and methods

### 2.1 Research design and participants

This cross-sectional quantitative study employed structural equation modeling to test the proposed serial mediation model. The study was conducted at eight vocational undergraduate universities in Hebei Province, China, utilizing a stratified cluster sampling design to capture a broad cross-section of the vocational student population. Each university represented a cluster. Within each cluster, students were stratified by academic year (freshman through senior) and major discipline, then intact classes were randomly sampled from each stratum to ensure representation across different year levels and fields of study.

A total of 1,870 students were invited to participate in an online questionnaire survey. After data-quality screening, 1,533 valid responses remained, meaning that 337 responses (18.1% of invited participants) were excluded. The screening criteria were applied as a combined data-quality procedure because individual cases could meet more than one criterion. Therefore, the exclusion process is reported transparently as a total exclusion count with clearly specified decision rules rather than as mutually exclusive category counts that could overstate precision.

Responses were excluded if they met one or more of the following criteria: (a) implausibly short completion time, defined as less than 120 s for the full questionnaire; (b) highly patterned responding, including long strings of identical answers across conceptually distinct items; (c) more than 10% missing responses; or (d) failure of embedded attention-check items (for example, an instruction to select a specific response option). The 120-s threshold was used to identify responses unlikely to reflect meaningful reading of the questionnaire, the long-string criterion targeted straight-lining and inattentive response patterns, and the 10% missingness threshold was selected to limit bias from incomplete cases while avoiding unnecessary loss of otherwise usable data. These procedures are consistent with recommendations for identifying careless responses and insufficient effort responding in online survey research (Meade and Craig, 2012; DeSimone et al., 2015) and with the need for careful data screening before structural equation modeling. The final valid sample was *N* = 1,533, reflecting an effective response rate of 81.9%.

Table 1 presents detailed demographic characteristics of participants. The sample demonstrated balanced distribution across gender and grade levels, and the urban–rural ratio aligned with the broader vocational student population in the province.

**Table 1:** Demographic characteristics of participants (*N* = 1,533).

Characteristic	*n* (%)	Mean (SD) / Range
Gender		
Male	786 (51.3)	
Female	747 (48.7)	
Academic year		
First year	518 (33.8)	
Second year	445 (29.0)	
Third year	369 (24.1)	
Fourth year	201 (13.1)	
Major field		
Science/Engineering	590 (38.5)	
Humanities/Social sciences	540 (35.2)	
Management/Economics	311 (20.3)	
Arts/others	92 (6.0)	
Hometown background		
Urban	897 (58.5)	
Rural	636 (41.5)	
Age (years)		19.4 (1.3); Range: 18–23
Teacher support (PTS)		3.83 (0.72); Range: 1.00–5.00
Self-Efficacy (CSSE)		3.24 (0.68); Range: 1.00–5.00
Speaking anxiety (CSA)		3.09 (0.74); Range: 1.00–5.00
Silence tendency (CST)		3.16 (0.71); Range: 1.00–5.00

The final sample comprised 51.3% male (*n* = 786) and 48.7% female (*n* = 747), with ages ranging from 18 to 23 years (M = 19.4, SD = 1.3). All four undergraduate year levels were represented: approximately 33.8% were first-year students (*n* = 518), 29.0% second-year (*n* = 445), 24.1% third-year (*n* = 369), and 13.1% fourth-year (*n* = 201). Students majored in various fields: about 38.5% in Sciences or Engineering (*n* = 590), 35.2% in Humanities or Social Sciences (*n* = 540), 20.3% in Management or Economics (*n* = 311), and 6.0% in Arts or other fields (*n* = 92). Additionally, 58.5% of participants reported urban hometowns (*n* = 897) and 41.5% rural areas (*n* = 636). Overall, the sample’s gender balance, disciplinary mix, and urban–rural background support the contextual adequacy of the sample for examining vocational undergraduates in this provincial setting.

### 2.2 Theoretical framework

#### 2.2.1 Classroom ecology and teacher support as contextual foundation

A classroom ecological perspective emphasizes that student behavior cannot be understood in isolation from surrounding environment (Leicht and Heiss, 2018). Drawing on Bronfenbrenner’s bio-ecological model of human development (Kioupi and Voulvoulis, 2019), the classroom functions as a complex micro-ecosystem wherein teachers play central regulatory roles, shaping emotional and academic climates. In this ecosystem, perceived teacher support (PTS) constitutes a critical environmental resource encompassing multiple dimensions: emotional support (caring for and respecting students as individuals), instrumental support (providing help, feedback, and academic scaffolding), and autonomy support (valuing students’ perspectives and encouraging choice) (Bronfenbrenner, 1979; El Zaatari and Maalouf, 2022).

When students perceive classroom climates as supportive and non-threatening, threat-monitoring systems can be down-regulated, freeing cognitive resources for engagement and risk-taking in speaking (Skinner et al., 2008). Indeed, in the post-2020 educational landscape marked by pandemic disruptions and heightened student stress, psychological safety provided by supportive teachers has become a recognized prerequisite for student participation (Forbes et al., 2011; Roorda et al., 2011).

Importantly, teacher support’s impact is not necessarily direct or uniform across all contexts. Some studies have found significant direct effects of perceived support on students’ classroom engagement (Xu and Qi, 2019), whereas others indicate this relationship is largely indirect, mediated by students’ internal beliefs and emotional states (Fredricks et al., 2004; Chiu, 2023). In the specific context of defensive silence, teacher support functions as a distal antecedent, creating necessary conditions for change through safe, encouraging atmospheres, while proximal drivers of speaking behavior remain the student’s own confidence and anxiety levels (Jiang et al., 2019; Tauchid, 2025).

#### 2.2.2 Social cognitive theory: the mediating role of self-efficacy

Bandura’s Social Cognitive Theory (SCT) offers a lens for understanding how external support translates into internal agency. SCT posits that “people’s level of motivation, affective states, and actions are based more on what they believe than on what is objectively true”. Central to SCT is self-efficacy—one’s belief in capability to organize and execute actions required to manage prospective situations (Bandura, 2002). In classroom participation contexts, perceived teacher support is hypothesized to enhance students’ speaking self-efficacy via multiple pathways corresponding to Bandura’s four classic sources of efficacy information (Locke, 1997):

*Mastery experiences*: Supportive teachers scaffold class activities enabling students to achieve small “wins” in speaking, building success track records. These mastery experiences represent Bandura’s most powerful efficacy source; accumulated successful speaking attempts provide tangible competence evidence, helping students develop more stable confidence in speaking (Schunk and DiBenedetto, 2020).

*Vicarious experience*: In supportive classroom climates, when students speak up, teachers react with respect and positive reinforcement. Observing peers speak without suffering negative consequences signals to others that speaking is safe and feasible—a vicarious efficacy boost (Maulidha and Tiatri, 2023). Teachers managing class discussions well effectively model inclusive environments, encouraging silent observers to imagine themselves participating.

*Verbal persuasion*: Teachers high in support frequently express confidence in students’ abilities. This verbal encouragement from respected authority can persuade students to attempt speaking tasks they might otherwise avoid. Over time, such messages can internalize into self-beliefs (Usher and Pajares, 2008). Persuasion alone is weaker than actual success; its influence is greatest when paired with genuine mastery experiences maintaining credibility (Schunk, 1987).

*Physiological/Affective States*: Supportive teachers cultivate calm, respectful classroom climates helping students reappraise their physiological arousal. A caring teacher might normalize nervousness. By reducing tension and framing arousal as excitement rather than panic, teachers help students keep anxiety in check, indirectly bolstering efficacy (Zientek et al., 2019).

Through these mechanisms, higher teacher support is expected to be associated with higher classroom speaking self-efficacy. Students begin believing “I can express my ideas” when environments continually signal acceptance and provide structured success opportunities. Higher efficacy is expected to support approach-oriented behavior (asking questions, volunteering answers), whereas lower efficacy is expected to be associated with avoidance-oriented behavior (staying silent) (Meng and Zhang, 2023; Samaila et al., 2024).

#### 2.2.3 Control-value theory: the mediating role of anxiety

Pekrun’s Control-Value Theory (CVT) of achievement emotions explains how anxiety is associated with achievement-related appraisals and performance (Pekrun and Goetz, 2023). According to CVT, anxiety is a prospective, outcome-focused emotion arising from two appraisals: value (the student highly values the outcome, such as maintaining “face” or gaining approval) and control (the student feels low control over achieving positive outcomes). In the current context, a student might greatly value answering correctly to gain praise or avoid embarrassment yet feel low control due to lacking confidence in their knowledge or speaking ability. This combination represents a condition likely to increase anxiety (Pajares, 1996).

Self-efficacy provides the primary appraisal of control: a student with high classroom speaking self-efficacy (CSSE) feels capable of handling speaking tasks, mitigating anxiety. Conversely, low CSSE means the student perceives little control; thus, even when highly valuing participation or reputation, that high value only heightens anxiety when coupled with low perceived control (Pekrun, 2024; Pekrun and Goetz, 2024).

Anxiety, once triggered, has several detrimental effects in classrooms. It creates avoidance motivation—students focus on avoiding failure or humiliation rather than seeking success (Goetz et al., 2008). It also interferes with cognitive processing: worry and self-focus consume working memory resources, making it harder to formulate and articulate thoughts spontaneously (Dewaele and Li, 2021). Physically, anxiety manifests in somatic symptoms (sweaty palms, pounding heart, trembling voice) making speaking even more challenging. High anxiety directly encourages silence as an immediate coping mechanism to evade feared outcomes (Eysenck et al., 2007; Putwain and Symes, 2011).

**Figure 1:**
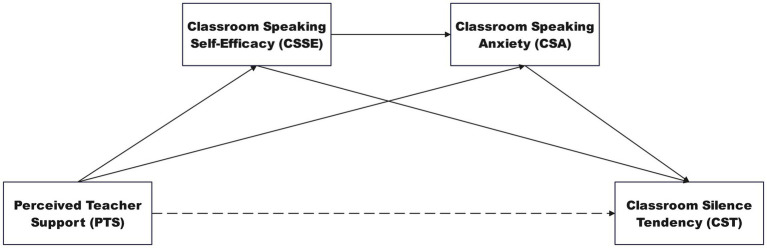
The hypothesized serial mediation model linking perceived teacher support to classroom silence. PTS, Perceived Teacher Support; CSSE, Classroom Speaking Self-Efficacy; CSA, Classroom Speaking Anxiety; CST, Classroom Silence Tendency. Solid lines indicate hypothesized significant paths; dashed line represents hypothesized non-significant direct effect.

Critically, self-efficacy and anxiety are inversely linked in this framework. Efficacy acts as a protective shield against anxiety by instilling control sense. If a student truly believes “I can do this,” perceived threat is reduced and anxiety subsides. Low efficacy amplifies threat (e.g., failure risk or losing face) and thereby fuels anxiety (Azizpour and Gholami, 2022). This relationship is empirically borne out; recent studies found that Chinese students perceiving greater teacher support had lower speaking anxiety primarily because support boosted self-efficacy and positive mindset (Schunk and DiBenedetto, 2020; Forsblom et al., 2022) (see Figure 1).

### 2.3 Hypotheses

Based on the theoretical integration of social cognitive theory and control-value theory, this study proposes a serial mediation model wherein perceived teacher support is associated with classroom silence indirectly through students’ speaking self-efficacy and speaking anxiety. Specifically:

*H1*: Higher perceived teacher support is associated with higher classroom speaking self-efficacy (PTS → CSSE).

*H2*: Higher speaking self-efficacy is associated with lower speaking anxiety (CSSE → CSA).

*H3*: Lower speaking anxiety is associated with lower tendency to remain silent (CSA → CST).

*H4*: Higher speaking self-efficacy is associated with lower classroom silence tendency (CSSE → CST), independent of anxiety.

*H5*: The association between perceived teacher support and classroom silence will be statistically accounted for by speaking self-efficacy and speaking anxiety in the specified serial mediation model. The serial indirect pathway is expected to make the largest contribution among the tested indirect pathways, while the direct path from perceived teacher support to classroom silence is expected to be non-significant after the mediators are included.

### 2.4 Instruments

Data were collected via an online survey battery titled “Classroom Participation Phenomena Survey.” This was not a newly developed single psychometric instrument. Rather, it was a compiled questionnaire consisting of adapted and localized scales corresponding to the four constructs in the proposed model: perceived teacher support, classroom speaking self-efficacy, classroom speaking anxiety, and classroom silence tendency. The construct-level structure of the source scales was preserved, while item wording was adjusted to fit the Chinese vocational higher education context and the specific situation of speaking in class.

All items were rated on 5-point Likert-type scales (1 = Strongly Disagree to 5 = Strongly Agree). The final analytic questionnaire included 28 retained items: 8 items for perceived teacher support, 4 items for classroom speaking self-efficacy, 8 items for classroom speaking anxiety, and 8 items for classroom silence tendency. The classroom speaking self-efficacy section was initially built from an 8-item pool, but four items were removed after confirmatory factor analysis because their loadings were below the recommended 0.70 threshold. Each scale was translated and back-translated (English-Chinese-English) to ensure semantic accuracy. The survey was pilot-tested with 50 vocational students to evaluate clarity, wording, and cultural appropriateness. The pilot led to minor wording refinements but no changes to the intended construct structure. Because the pilot sample was intended for clarity checking rather than stable psychometric estimation, reliability was evaluated in the full study sample. McDonald’s omega values ranged from 0.831 to 0.901 across constructs, indicating good to excellent internal consistency and supporting the adequacy of the measures for the mediation analysis.

#### 2.4.1 Perceived teacher support (PTS)

Students’ perceptions of teacher support were measured using an 8-item scale adapted from Wentzel’s pedagogical caring scale (Wentzel, 1997), with wording localized for the Chinese vocational context. This scale captures how approachable, encouraging, and understanding students perceive their teacher to be. Sample items include: “My teacher creates a classroom atmosphere where it is safe to make mistakes,” and “The teacher listens carefully when I express my opinion.” Higher scores indicate greater perceived teacher support. In the present study, internal reliability was excellent (*ω* = 0.901, 95% CI [0.89, 0.91]).

#### 2.4.2 Classroom speaking self-efficacy (CSSE)

Students’ confidence in their ability to speak up in class was assessed using a scale constructed following Bandura’s guidelines for self-efficacy measurement (Bandura, 1997) and drawing on prior adaptations for classroom speaking contexts (Liu et al., 2025). An initial item pool of eight items was subjected to confirmatory factor analysis; four items (items 1, 3, 4, and 6) were removed due to factor loadings below the recommended 0.70 threshold, yielding a final 4-item scale. Items asked students to rate confidence in performing various speaking-related behaviors under typical classroom conditions. Sample items include: “I am confident I can express my ideas clearly during class discussions,” and “I can respond to the teacher’s questions even if they are difficult.” Higher scores indicate greater self-efficacy in classroom communication. The final 4-item scale demonstrated robust reliability (*ω* = 0.831, 95% CI [0.82, 0.85]).

#### 2.4.3 Classroom speaking anxiety (CSA)

Speaking-related anxiety was measured using an adapted version of the Foreign Language Classroom Anxiety Scale (Horwitz et al., 1986). Since the context involves speaking in native language (Mandarin) rather than foreign language, items were modified to refer to any form of public speaking or responding in class. This 8-item adapted scale emphasizes fear of negative evaluation and somatic symptoms of anxiety in classroom speaking situations. Sample items include: “I feel my heart pounding when I am about to be called on to speak,” and “I worry that other students will laugh at me if I speak up.” Higher scores indicate greater speaking anxiety. Internal consistency was high (*ω* = 0.873, 95% CI [0.86, 0.88]).

#### 2.4.4 Classroom silence tendency (CST)

The outcome variable, tendency toward classroom silence, was measured with a 8-item scale developed to assess frequency and intentionality of staying silent in class (Hu, 2021). This instrument distinguishes defensive or avoidance-driven silence from neutral or reflective silence, focusing on avoidance-motivated behaviors. Sample items include: “I never volunteer to answer questions in class,” and “I sometimes pretend to be busy reading or writing so the teacher won’t call on me.” Higher scores indicate stronger habitual inclination to refrain from speaking. This scale showed high reliability (*ω* = 0.883, 95% CI [0.87, 0.89]).

For all constructs, scales were scored by averaging items so composite scores remained on the original 1–5 scale. By this coding, higher scores on PTS and CSSE indicate more perceived support and higher confidence, respectively, whereas higher scores on CSA and CST indicate greater anxiety and stronger tendency to remain silent.

### 2.5 Data collection procedure

Data collection was conducted mid-semester (weeks 8–10 of a 16-week term) to ensure students had sufficient exposure to instructors to form stable perceptions of teacher support and to mitigate unusual start-of-term or end-of-term effects. After obtaining approval from the university ethics review board and permissions from each institutional administration, a local project coordinator at each university facilitated recruitment. The survey link was distributed to students either during class sessions (by instructors projecting a QR code or URL) or via universities‘online learning platforms.

Participation was voluntary and anonymous. Students were informed that there were no right or wrong answers and that responses would be used solely for research aimed at improving teaching practices. Confidentiality assurances aimed to reduce evaluation apprehension. To minimize common method bias and encourage honest responses, several procedural remedies were implemented (Podsakoff et al., 2024). First, the survey emphasized anonymity and confidentiality. Second, questionnaire section order was counterbalanced and interspersed with unrelated filler items, reducing likelihood of participants inferring study hypotheses or answering in patterned ways. Third, attention-check questions (e.g., “Select ‘Agree’ for this item”) were embedded to identify inattentive respondents; such cases were removed during data cleaning.

### 2.6 Statistical analysis

All quantitative analyses were conducted using IBM SPSS 26 and SmartPLS 4.0. A two-step analytical strategy was adopted, consistent with recommendations for structural equation modeling: first validating the measurement model, then testing the structural model and mediation hypotheses (Cheung et al., 2024).

#### 2.6.1 Preliminary analyses

Data were inspected for anomalies. Descriptive statistics and Pearson correlations were computed for all key variables (PTS, CSSE, CSA, CST) to understand basic relationships and check for out-of-range values or severe non-normality. Univariate normality was evaluated by examining skewness and kurtosis for each variable; all distributions had skewness and kurtosis within ±1.0, indicating no significant departures from normality, confirming that univariate distributions were well-behaved, which is consistent with the distributional assumptions of the planned PLS-SEM analyses (Hair and Alamer, 2022).

#### 2.6.2 Common method variance assessment

Given the cross-sectional, self-report nature of data, potential common method variance (CMV) was evaluated using Harman’s single-factor test (Bozionelos and Simmering, 2022). An exploratory factor analysis of all survey items (unrotated) revealed four distinct factors with eigenvalues greater than 1. The first factor accounted for only 27.2% of variance, well below the 40% threshold often used as a heuristic criterion for substantial common method variance. This suggests no single general factor dominates covariance in the data, reducing CMV concerns. Additionally, a more stringent Variance Inflation Factor (VIF) collinearity assessment was conducted following the full collinearity approach recommended for PLS-SEM applications (Sarstedt et al., 2021; Podsakoff et al., 2024). Each construct was treated alternately as a predictor and criterion variable in auxiliary regressions involving all remaining constructs, with the resulting VIF values serving as CMV indicators. As shown in Table 2, VIF values for all four constructs ranged narrowly from 1.42 (PTS) to 1.88 (CST), all well below the conservative threshold of 3.3 (Ringle et al., 2023).

**Table 2:** Descriptive statistics, reliabilities, and correlation matrix (*N* = 1,533).

Variable	M	SD	α	ω	1	2	3	4	VIF
1. PTS	3.83	0.72	0.895	0.901	**(0.810)**				1.42
2. CSSE	3.24	0.68	0.822	0.831	0.529***	**(0.742)**			1.58
3. CSA	3.09	0.74	0.868	0.873	−0.108***	−0.352***	**(0.787)**		1.87
4. CST	3.16	0.71	0.879	0.883	−0.063*	−0.342***	0.641***	**(0.800)**	1.88

PTS, Perceived Teacher Support; CSSE, Classroom Speaking Self-Efficacy; CSA, Classroom Speaking Anxiety; CST, Classroom Silence Tendency. Diagonal values in parentheses represent square roots of AVE. *p < 0.05, **p < 0.01, ***p < 0.001. VIF, Collinearity Variance Inflation Factor. All VIF values < 3.3 threshold, indicating no significant common method bias. Bold values on the diagonal (in parentheses) are the square roots of the AVE.

#### 2.6.3 Measurement model evaluation

Partial Least Squares Structural Equation Modeling (PLS-SEM) was employed as the primary analytical approach due to several advantages for this study’s objectives (Cheung et al., 2024; Sathyanarayana and Mohanasundaram, 2024). PLS-SEM is particularly suitable when: (a) the research model is complex with multiple mediators, (b) the focus is on prediction rather than theory confirmation alone, (c) the data distribution may deviate from multivariate normality, and (d) formative measurement models may be considered. Additionally, PLS-SEM provides robust estimates with relatively smaller sample sizes and is less sensitive to data distribution assumptions compared to covariance-based SEM (Hair and Alamer, 2022).

The measurement model was evaluated using established criteria (Reinartz et al., 2009; Hair et al., 2012). For reflective constructs, internal consistency reliability was assessed using Cronbach’s alpha (*α* > 0.70) and composite reliability (CR > 0.70). Convergent validity was examined through average variance extracted (AVE > 0.50) and standardized factor loadings (*λ* > 0.70). Discriminant validity was assessed using the Fornell-Larcker criterion (Sarstedt et al., 2021), wherein the square root of each construct’s AVE should exceed its correlations with other constructs, and the Heterotrait-Monotrait ratio (HTMT < 0.85) (Wu et al., 2024).

#### 2.6.4 Structural model testing and mediation analysis

After establishing satisfactory measurement model properties, the structural equation model representing hypothesized serial mediation relationships was specified. Teacher support (PTS) was modeled as the exogenous predictor, classroom silence (CST) as the ultimate outcome, and speaking self-efficacy (CSSE) and speaking anxiety (CSA) as sequential mediators. Direct paths were included from PTS to each mediator (PTS → CSSE, PTS → CSA) and to the outcome (PTS → CST), as well as paths from CSSE to CSA and CST, and from CSA to CST.

Model fit was evaluated using standard indices: Standardized Root Mean Square Residual (SRMR < 0.08) and Normed Fit Index (NFI > 0.90). Predictive relevance was assessed using Stone-Geisser’s Q^2^ values (Q^2^ > 0), calculated through blindfolding procedures with omission distance of 7 (Rönkkö and Cho, 2022). Effect sizes were evaluated using Cohen’s f^2^ values, where f^2^ ≥ 0.02, f^2^ ≥ 0.15, and f^2^ ≥ 0.35 represent small, medium, and large effects, respectively (Hair et al., 2012).

To rigorously test mediation hypotheses, bias-corrected bootstrapping procedures (5,000 resamples) were employed to construct 95% confidence intervals for all indirect, direct, and total effects (Tibbe and Montoya, 2022). An indirect effect was considered statistically significant if its 95% confidence interval did not include zero. The proportion of total effect of PTS on CST mediated by each specific pathway was calculated to compare relative contributions of simple versus serial mediations.

Given the potential for suppressor effects in complex mediation models—wherein inclusion of one mediator may cause the path coefficient through another mediator to differ in sign from its bivariate counterpart—specific indirect effects were interpreted in terms of their bootstrapped estimates rather than products of structural path coefficients. A positive indirect effect for a given pathway does not necessarily imply a positive zero-order association between predictor and outcome (Lethbridge et al., 2024). Throughout the manuscript, terms such as direct effect and indirect effect refer to statistical estimates within the specified cross-sectional SEM model and should not be interpreted as evidence of causal effects.

#### 2.6.5 Multigroup analysis

Given roughly equal numbers of male and female students in the sample, measurement invariance across genders was examined using multigroup analysis in PLS-SEM. The MICOM procedure was conducted, testing configural invariance (same construct specification across groups), compositional invariance (same composite scores across groups), and equality of composite mean values and variances (Yu et al., 2024). Following establishment of measurement invariance, path coefficient differences between groups were tested using permutation tests.

## 3 Results

### 3.1 Descriptive statistics and preliminary analyses

Table 2 presents descriptive statistics, reliability coefficients, and intercorrelations for all study variables. On average, students reported moderately high teacher support and self-efficacy levels [M(PTS) = 3.83, SD = 0.72; M(CSSE) = 3.24, SD = 0.68], whereas mean anxiety was closer to midpoint [M(CSA) = 3.09, SD = 0.74] and mean silence tendency was slightly above neutral [M(CST) = 3.16, SD = 0.71]. These averages suggest that, in general, students felt teachers were quite supportive and had fair degrees of confidence, yet many still experienced some anxiety and inclination to stay quiet.

In terms of zero-order correlations, perceived teacher support had a small but statistically significant negative correlation with classroom silence (*r* = −0.063, *p* < 0.05), suggesting students feeling more supported by teachers tended to report being slightly less silent. However, the weakness of this correlation hints that simply having a supportive teacher does not automatically translate into an active class—an initial indication explored through mediation analysis. Teacher support was much more strongly correlated with speaking self-efficacy (*r* = 0.529, *p* < 0.01), indicating supportive teachers seem highly effective at building students’ confidence to speak. Self-efficacy showed significant negative correlations with both anxiety and silence (*r*(CSSE, CSA) = −0.352; *r*(CSSE, CST) = −0.342, *p* < 0.01 for both), suggesting confidence is associated with less fear and less withdrawal. As expected, classroom speaking anxiety had a strong positive correlation with silence (*r* = 0.641, *p* < 0.01): the more anxious students felt about speaking, the more likely they were to keep quiet.

### 3.2 Measurement model assessment

Table 3 presents comprehensive psychometric properties of the measurement model. All standardized factor loadings met or exceeded the recommended 0.70 threshold (range: 0.703–0.841, *p* < 0.001), indicating that indicators adequately represented their respective latent constructs (Hair and Alamer, 2022). Internal consistency was excellent across all measures, with Cronbach’s alpha values ranging from 0.822 to 0.895 and composite reliability values from 0.843 to 0.903, all surpassing recommended thresholds of 0.70 (Hair and Alamer, 2022).

**Table 3:** Measurement model: factor loadings, reliability, and convergent validity (*N* = 1,533).

Construct/Item	Loading	*t*-value	α	ω	CR	AVE
Perceived teacher support (PTS)			0.895	0.901	0.903	0.656
PTS1	0.800	32.43				
PTS2	0.788	31.79				
PTS3	0.821	33.82				
PTS4	0.833	34.34				
PTS5	0.757	28.99				
PTS6	0.823	34.06				
PTS7	0.822	33.93				
PTS8	0.833	34.37				
Classroom speaking self-efficacy (CSSE)			0.822	0.831	0.843	0.551
CSSE2	0.703	29.84				
CSSE5	0.784	34.92				
CSSE7	0.760	32.55				
CSSE8	0.720	30.38				
Classroom speaking anxiety (CSA)			0.868	0.873	0.881	0.619
CSA1	0.814	34.36				
CSA2	0.799	33.77				
CSA3	0.794	33.54				
CSA4	0.743	31.64				
CSA5	0.747	31.90				
CSA6	0.820	35.17				
CSA7	0.775	32.81				
CSA8	0.800	33.90				
Classroom silence tendency (CST)			0.879	0.883	0.889	0.640
CST1	0.807	34.41				
CST2	0.824	35.04				
CST3	0.787	33.4				
CST4	0.779	33.05				
CST5	0.792	33.68				
CST6	0.769	32.81				
CST7	0.802	34.17				
CST8	0.841	35.78				

All factor loadings significant at ***p < 0.001. CR, composite reliability; AVE, average variance extracted; α, Cronbach’s Alpha; ω, McDonald’s Omega. Composite reliability and AVE values meet or exceed recommended thresholds (CR > 0.70; AVE > 0.50), supporting measurement model adequacy.

Convergent validity was strongly supported, as Average Variance Extracted (AVE) for all constructs exceeded 0.50 (range: 0.551–0.656), indicating that constructs explain more than half of the variance in their indicators. Additionally, all composite reliability values exceeded corresponding AVE values, further supporting convergent validity (Hair and Alamer, 2022).

Discriminant validity was assessed using multiple criteria. Table 4 presents the Fornell-Larcker criterion results, wherein the square root of each construct’s AVE (shown on diagonal) exceeded its correlations with other constructs. Furthermore, Heterotrait-Monotrait (HTMT) ratios were computed, with all values falling below the stringent threshold of 0.85 (range: 0.067–0.708), which is more demanding than the standard 0.90 criterion, confirming discriminant validity (Sarstedt et al., 2022).

**Table 4:** Discriminant validity assessment: Fornell–Larcker criterion and HTMT ratios (*N* = 1,533).

Construct	PTS	CSSE	CSA	CST
PTS	**0.810**	0.587	0.119	0.067
CSSE	0.529	**0.742**	0.389	0.378
CSA	−0.108	−0.352	**0.787**	0.708
CST	−0.063	−0.342	0.641	**0.800**

Diagonal elements (in bold) represent square roots of AVE. Off-diagonal elements below diagonal show inter-construct correlations; above diagonal shows HTMT ratios. All HTMT values < 0.85, confirming discriminant validity.

### 3.3 Structural model results

The hypothesized structural model demonstrated excellent fit to the data. The Standardized Root Mean Square Residual (SRMR) was 0.046, well below the threshold of 0.08, and the Normed Fit Index (NFI) was 0.958, exceeding the recommended cutoff of 0.90 (Schuberth et al., 2023). These indices collectively indicate that the proposed model adequately represents the observed data structure.

**Figure 2:**
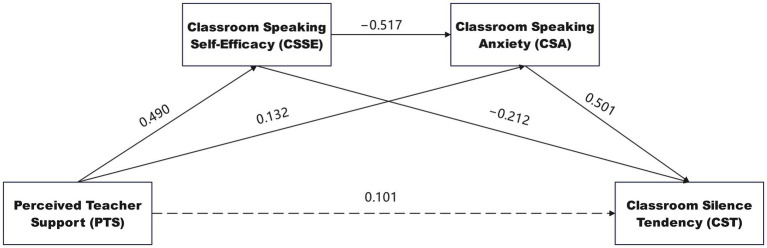
Tested serial mediation model with standardized path coefficients and significance levels. Standardized coefficients shown. Solid lines indicate significant paths (*p* < 0.001); dashed line represents non-significant direct effect. PTS, perceived teacher support; CSSE, classroom speaking self-efficacy; CSA, classroom speaking anxiety; CST, classroom silence tendency. Path coefficients and *t*-values derived from PLS-SEM with 5,000 bootstrap resamples; *R*^2^ values for endogenous constructs are reported in Table 5.

Figure 2 presents the tested serial mediation model with standardized path coefficients. As hypothesized, perceived teacher support showed a strong positive association with classroom speaking self-efficacy (*β* = 0.490, *t* = 24.87, *p* < 0.001), supporting H1. This finding indicates that students perceiving higher levels of teacher support reported significantly greater confidence in their ability to participate verbally in class. The effect size was large (*f*^2^ = 0.364), suggesting teacher support accounts for substantial variance in speaking self-efficacy.

Classroom speaking self-efficacy was strongly negatively associated with speaking anxiety (*β* = −0.517, *t* = 21.43, *p* < 0.001), supporting H2. Students with greater belief in their ability to contribute in class experienced substantially less anxiety about doing so. The effect size was large (*f*^2^ = 0.455), underscoring self-efficacy’s critical role in reducing anxiety. Critically, teacher support showed a weak but statistically significant positive direct association with speaking anxiety (*β* = 0.132, *t* = 3.76, *p* < 0.001, *f*^2^ = 0.011). This pattern is consistent with a possible suppressor-like effect and is interpreted further in the Discussion.

Consistent with H3, speaking anxiety was substantially and positively associated with classroom silence tendency (*β* = 0.501, *t* = 21.51, *p* < 0.001), with large effect size (*f*^2^ = 0.345). This coefficient was the largest among direct paths, highlighting anxiety as a key proximal correlate of whether students speak or withdraw. Higher anxiety was associated with a higher propensity for students to remain silent, supporting the conceptualization of silence as an avoidance strategy to evade distress of speaking.

Supporting H4, even after accounting for anxiety, self-efficacy retained a direct negative association with silence (*β* = −0.212, *t* = 8.87, *p* < 0.001), with medium effect size (*f*^2^ = 0.062). Beyond its indirect influence via reducing anxiety, higher self-efficacy was independently associated with less silent behavior, suggesting self-efficacy may also encourage proactive engagement through additional routes—for example, confident students might speak up from eagerness or habit, even without substantial anxiety.

Critically, supporting H5, the direct path from teacher support to classroom silence was non-significant (*β* = 0.101, *t* = 0.68, *p* = 0.495, *f*^2^ < 0.001). In statistical terms, this indicates that once students’ self-efficacy and anxiety were included in the model, perceived teacher support did not retain a detectable direct pathway to classroom silence. The zero-order association between supportive teaching and lower student silence was therefore accounted for by the mediating psychological variables in the specified model.

Table 5 presents the model’s explained variance (R^2^) and predictive relevance (Q^2^) for endogenous constructs. The model explained 26.7% of variance in classroom speaking self-efficacy, 31.8% in speaking anxiety, and 52.1% in classroom silence tendency. All Q^2^ values exceeded zero (range: 0.214–0.411), indicating the model has adequate predictive relevance for all endogenous constructs.

**Table 5:** Explained variance (*R*^2^) and predictive relevance (*Q*^2^) of endogenous constructs.

Construct	*R* ^2^	*R*^2^ Adjusted	*Q* ^2^	Interpretation
CSSE	0.267	0.265	0.214	Moderate predictive power
CSA	0.318	0.316	0.251	Moderate predictive power
CST	0.521	0.520	0.411	Substantial predictive power

R^2^, Coefficient of determination; Q^2^, Stone-Geisser’s predictive relevance statistic calculated via blindfolding procedure (omission distance = 7). All Q^2^ > 0 indicates adequate predictive relevance.

### 3.4 Mediation analysis

Table 6 presents the comprehensive decomposition of direct, indirect, and total statistical estimates in the serial mediation model. The total indirect estimate from teacher support to classroom silence was substantial and statistically significant (*β* = −0.166, 95% CI [−0.194, −0.139], *p* < 0.001), while the direct path remained non-significant (*β* = 0.101, 95% CI [−0.189, 0.391], *p* = 0.495). This pattern is consistent with a fully mediated statistical model within the tested cross-sectional SEM framework.

**Table 6:** Decomposition of effects: direct, indirect, and total effects of teacher support on classroom silence.

Path	Effect	*β*	*SE*	*t*	95% CI		*p*-value
					Lower	Upper	
Total effect (PTS → CST)		−0.065	0.031	2.1	−0.126	−0.004	0.036
Direct effect		0.101	0.148	0.68	−0.189	0.391	0.495
Total indirect effect		−0.166	0.014	11.86	−0.194	−0.139	<0.001
Specific indirect effects							
Path 1: PTS → CSSE → CST	Simple via efficacy	−0.104	0.014	7.43	−0.132	−0.077	<0.001
Path 2: PTS → CSA → CST	Simple via anxiety	0.066	0.012	5.5	0.043	0.090	<0.001
Path 3: PTS → CSSE → CSA → CST	Serial mediation	−0.127	0.012	10.58	−0.151	−0.104	<0.001

Standardized estimates based on 5,000 bootstrap resamples. CI, confidence interval. Indirect effects: Path 1 = PTS → CSSE → CST (simple mediation via self-efficacy); Path 2 = PTS → CSA → CST (simple mediation via anxiety); Path 3 = PTS → CSSE → CSA → CST (serial mediation via self-efficacy and anxiety). Total indirect effect = sum of all specific indirect effects.

Examining specific indirect pathways reveals three statistical routes linking teacher support with classroom silence in the specified model. The first pathway, simple mediation via self-efficacy (PTS → CSSE → CST), yielded a negative indirect estimate (*β* = −0.104, 95% CI [−0.132, −0.077], *p* < 0.001), accounting for approximately 35.0% of the total mediated estimate. This pattern suggests that the association between teacher support and lower silence tendency was partly carried through higher students’ confidence to speak, independent of anxiety.

The second pathway, simple mediation via anxiety alone (PTS → CSA → CST), demonstrated a positive indirect estimate (*β* = 0.066, 95% CI [0.043, 0.090], *p* < 0.001), representing a suppression-like pattern. This counterintuitive result suggests that although teacher support was negatively associated with silence overall, its direct statistical association with anxiety after controlling for efficacy was positive, possibly reflecting slightly greater performance pressure in some students. However, this small positive estimate (22.2% of the total absolute mediated variance, operating in the opposite direction to Paths 1 and 3) was outweighed by the confidence-related pathways, resulting in a net negative total indirect estimate.

The third pathway, serial mediation through both mediators (PTS → CSSE → CSA → CST), produced the strongest negative indirect estimate (*β* = −0.127, 95% CI [−0.151, −0.104], *p* < 0.001), accounting for approximately 42.8% of the total mediated estimate. This serial pathway represents the theorized statistical sequence in which higher teacher support is associated with higher self-efficacy, which is associated with lower anxiety and, in turn, lower silence tendency. The magnitude and precision of this estimate support the importance of the sequential psychological pathway in the specified model.

The non-significant direct path alongside a statistically significant total indirect estimate (*β* = −0.166, *p* < 0.001) is consistent with a fully mediated statistical model: within the specified SEM model, the association between teacher support and classroom silence was mainly carried through the two psychological mediators. Notably, the positive direct estimate (*β* = 0.101) combined with a larger negative total indirect estimate (*β* = −0.166) represents an inconsistent mediation pattern with suppression, wherein the inclusion of both mediators is essential for estimating the direction and magnitude of the association between teacher support and silence.

### 3.5 Subgroup analysis: heterogeneity in psychological pathways

**Figure 3:**
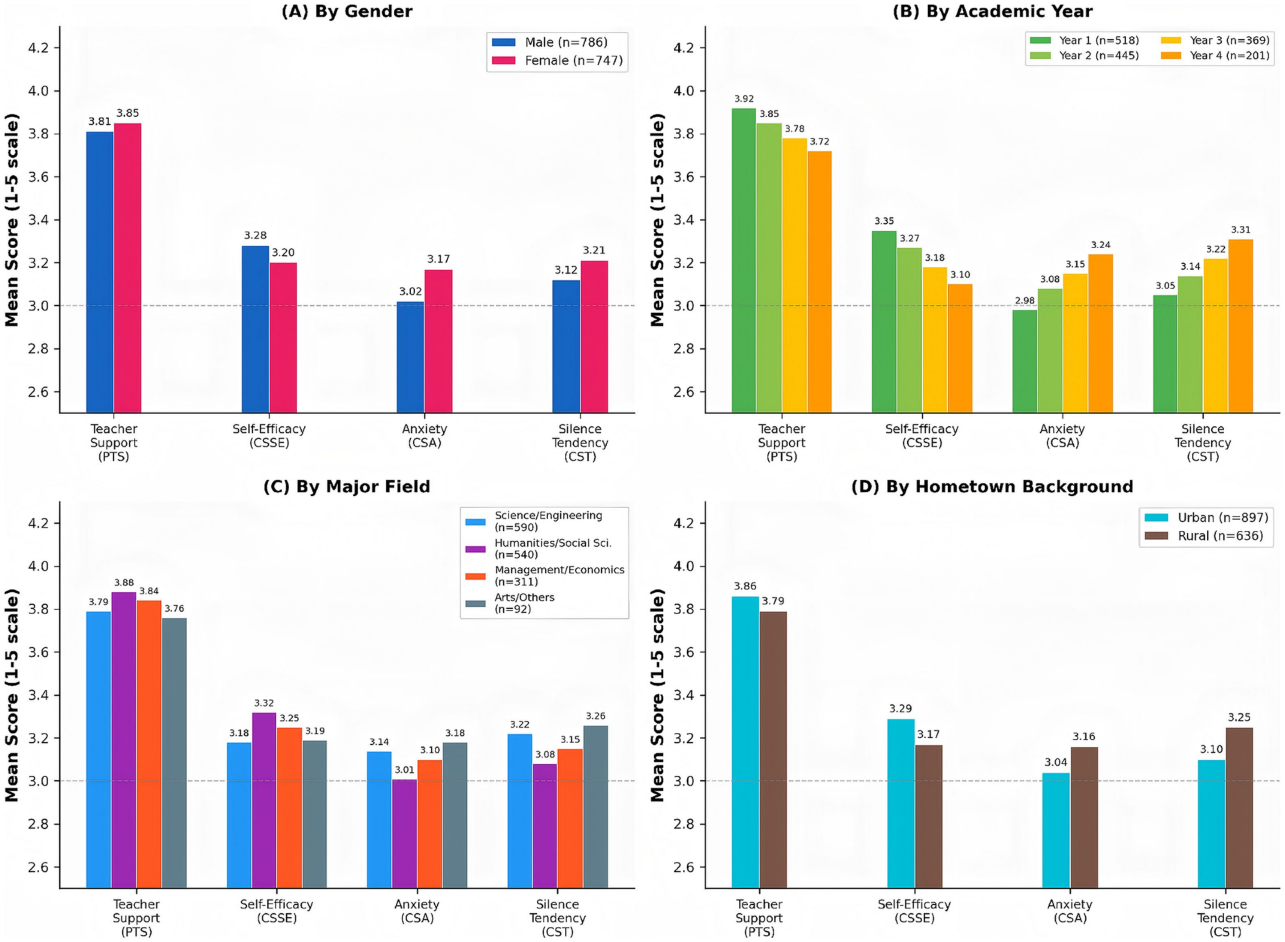
Mean scores of key variables across demographic subgroups (*N* = 1,533). PTS, perceived teacher support; CSSE, classroom speaking self-efficacy; CSA, classroom speaking anxiety; CST, classroom silence tendency. Panel **A** shows comparison by gender; panel **B** by academic year; panel **C** by major field; panel **D** by hometown background.

To provide nuanced understanding of how demographic factors may influence the psychological mechanisms underlying classroom silence, subgroup analyses were conducted examining mean differences across key demographic categories. While the multigroup structural analysis confirmed path invariance (indicating the psychological mechanisms operate similarly across subgroups), descriptive comparisons reveal important heterogeneity in baseline levels of study variables that have implications for targeted interventions. Figure 3 visualizes mean scores across demographic subgroups.

Panel A (By Gender) reveals subtle but noteworthy patterns: female students reported slightly higher teacher support perception (M = 3.85, SD = 0.70 vs. M = 3.81, SD = 0.74 for males) but also higher anxiety (M = 3.17, SD = 0.72 vs. M = 3.02, SD = 0.75) and greater silence tendency (M = 3.21, SD = 0.69 vs. M = 3.12, SD = 0.73). This pattern aligns with previous research indicating female students may be more sensitive to both supportive cues and evaluative threats in classroom settings.

Panel B (By Academic Year) reveals a concerning trajectory: as students progress through undergraduate years, they report decreasing teacher support perception, declining self-efficacy, increasing anxiety, and greater silence tendency. First-year students showed the most favorable profile (M(PTS) = 3.92, M(CSSE) = 3.35, M(CSA) = 2.98, M(CST) = 3.05), while fourth-year students exhibited the least favorable (M(PTS) = 3.72, M(CSSE) = 3.10, M(CSA) = 3.24, M(CST) = 3.31). This pattern suggests the “silent classroom” phenomenon may become entrenched over time, highlighting importance of early intervention.

Panel C (By Major Field) indicates students in Humanities and Social Sciences reported higher self-efficacy (M = 3.32) and lower silence tendency (M = 3.08) compared to counterparts in Science/Engineering (M(CSSE) = 3.18, M(CST) = 3.22) and Arts (M(CSSE) = 3.19, M(CST) = 3.26). This may reflect disciplinary differences in classroom communication expectations and teaching practices.

Panel D (By Hometown Background) shows rural-background students reported lower teacher support perception (M = 3.79 vs. M = 3.86), lower self-efficacy (M = 3.17 vs. M = 3.29), higher anxiety (M = 3.16 vs. M = 3.04), and greater silence tendency (M = 3.25 vs. M = 3.10) compared to urban-background students. This pattern suggests that students from less advantaged backgrounds may face additional psychological barriers to classroom participation.

### 3.6 Measurement invariance testing

To ensure that observed subgroup differences represent true differences in constructs rather than measurement artifacts, measurement invariance across gender was rigorously tested using the MICOM procedure in PLS-SEM. Table 7 presents results of the three-step invariance assessment.

**Table 7:** Measurement invariance assessment across gender (N_Male_ = 786, N_Female_ = 747).

Step	Construct	Correlation	Permutation p	2.5%/97.5% Quantile	Mean Diff	Variance Diff	Result
Step 1: Configural	All constructs	—	—	—	—	—	Established
Step 2: Compositional	PTS	0.999	0.412	—	—	—	Supported
	CSSE	0.999	0.381	—	—	—	Supported
	CSA	1	0.567	—	—	—	Supported
	CST	0.999	0.493	—	—	—	Supported
Step 3: Equal means/variances	PTS	—	—	−0.089	0.035	−0.012	Supported
	CSSE	—	—	−0.074	−0.019	0.008	Supported
	CSA	—	—	−0.095	−0.147	0.023	Supported
	CST	—	—	−0.082	−0.089	0.018	Supported

Step 1 = Configural Invariance (qualitative assessment of identical construct specification across groups); Step 2 = Compositional Invariance (correlation of composite scores = 1.000 and permutation *p*-value > 0.05); Step 3 = Equality of Composite Mean Values and Variances (2.5 and 97.5% quantiles contain original difference). Full measurement invariance established across all three steps for all constructs.

Step 1 (configural invariance) was established through qualitative assessment confirming identical construct specifications across male and female groups. Step 2 (compositional invariance) was supported for all constructs, with composite score correlations exceeding 0.999 and permutation test *p*-values > 0.05, indicating that composite scores have the same meaning across groups. Step 3 assessed equality of composite mean values and variances; for all constructs, the original mean and variance differences fell within the 95% confidence intervals from permutation, indicating no significant differences. Full measurement invariance was thus established, confirming that subsequent structural path comparisons reflect true differences rather than measurement inconsistencies.

Following establishment of measurement invariance, multigroup analysis comparing structural paths between male and female students revealed no significant differences in any of the hypothesized paths (all permutation *p*-values > 0.10). This indicates that the serial mediation mechanism operates similarly for both genders, despite the observed mean-level differences in study variables reported earlier.

## 4 Discussion

This investigation set out to examine the psychological architecture of the silent classroom in Chinese vocational higher education, addressing three key questions: (1) whether perceived teacher support is directly associated with students’ tendency to remain silent; (2) whether students’ speaking self-efficacy and speaking anxiety mediate the association between teacher support and silence; and (3) whether a sequential mediation mechanism links support to silence through efficacy and then anxiety. The empirical results clarify mechanisms underlying classroom silence. In brief, teacher support matters, but primarily through students’ internal confidence and anxiety pathways.

### 4.1 Indirect association between teacher support and classroom silence

One of the most important findings is that perceived teacher support demonstrated no significant direct statistical pathway to classroom silence (*β* = 0.101, *p* = 0.495). At face value, this result runs counter to the simple pedagogical assumption that caring, encouraging teachers automatically produce active, participatory classrooms. The data suggest a more nuanced pattern. A teacher can be kind and supportive, yet some classes may still remain silent if students do not translate that support into confidence and reduced evaluative fear. This interpretation is consistent with recent literature that treats support as a contextual resource whose benefits often depend on mediating psychological capacities rather than direct behavioral change.

Teacher support may create favorable classroom conditions, but it is unlikely to be associated with speaking behavior unless students internalize that support as confidence in their ability to participate. Encouragement and a positive classroom climate may be helpful, but they may not be sufficient to reduce silence unless students also develop confidence in their capacity to contribute effectively. In the context of Chinese vocational students, many of whom have experienced academic setbacks and doubts about their abilities, the default response even to supportive teachers may remain silence due to ingrained low academic self-concepts.

This finding echoes patterns observed in bystander intervention research. In bullying scenarios, simply providing reporting mechanisms or encouraging bystanders to intervene does not guarantee action; often, bystanders remain passive unless they feel confident in their ability to intervene (Putwain and Symes, 2011). Analogously, results suggest awareness of supportive environment (external support) is necessary but insufficient condition for breaking classroom silence. The sufficient condition is the student’s internalized sense of agency.

The positive indirect effect through anxiety alone should be interpreted cautiously. In the full model, perceived teacher support showed a small positive direct association with speaking anxiety after self-efficacy was included, whereas the overall indirect effect of teacher support on silence remained negative. This pattern is consistent with an inconsistent mediation or suppressor-like effect, suggesting that the association between teacher support and anxiety may be more complex than a simple protective pathway. One possible interpretation is that supportive teachers may, in some situations, also increase students’ awareness of participation expectations. However, this effect was small and was outweighed by the stronger negative indirect pathways operating through self-efficacy and through the sequential pathway from self-efficacy to anxiety.

### 4.2 Serial mediation through self-efficacy and anxiety

The findings are consistent with a theoretical account that combines Social Cognitive Theory and Control-Value Theory in explaining classroom participation. The significant serial pathway suggests that teacher support was associated with lower silence through higher speaking self-efficacy and lower speaking anxiety. This pattern is compatible with CVT’s premise that anxiety is closely tied to low perceived control: when students report stronger speaking self-efficacy, speaking situations may be appraised as more controllable and less threatening. In this interpretation, perceived teacher support is linked to stronger perceived speaking capability, which is in turn linked to lower anxiety and lower defensive silence.

Although the present model concerns a different population and a different outcome, its findings echo a structural regularity that has surfaced in adjacent educational literatures. Karacabey et al. (2025) found that transformational leadership reached teacher burnout largely indirectly—through organizational support and teacher resilience under workload demands—rather than through a direct path. A parallel pattern has been reported in studies of organizational change, where the leadership signal is carried by instructional strategy self-efficacy together with newer anxiety constructs such as AI anxiety (Atasoy and Özkul, 2026); and at the individual level, where pre-service teachers’ self-efficacy is associated with classroom management anxiety through self-directed learning (Özkul, 2025). What the present study adds is not a claim of equivalence with these populations, but evidence that the same internal architecture—contextual support, proximal psychological resource, anxiety, distal behavioural outcome—holds in a setting where the distal outcome is silence rather than burnout or organizational change, and where speaking itself carries cultural and institutional meaning that cannot be reduced to passive disengagement.

The strong negative link observed from self-efficacy to anxiety (*β* = −0.517) is particularly informative. It suggests that a substantial part of classroom speaking anxiety is associated with students’ perceived lack of control over speaking performance. This has practical implications for intervention. Traditional approaches often treat building confidence and reducing anxiety as separate goals, for example by using relaxation techniques or mindfulness to target anxiety while using public speaking drills to target performance ability. The present results are more consistent with an integrated approach: students who believe they can handle speaking tasks may experience less anxiety because the situation feels more controllable.

The cultural context of this study further reinforces this mechanism. In China’s collectivist and high-stakes educational environment, the prospect of losing face by making a public mistake can be a major source of anxiety. Higher self-efficacy may reduce the perceived interpersonal risk associated with speaking in public classroom settings. If students feel confident in their ability, the perceived risk to face may be lower because they expect to perform adequately. Conversely, low efficacy may amplify perceived face risk, triggering anxiety and contributing to silence. In this context, the serial mediation pattern may reflect a process in which supportive teaching is associated with greater speaking confidence, which is then associated with lower anxiety related to public participation and evaluation.

### 4.3 Interpreting the positive indirect effect through anxiety

An additional finding was the small positive indirect effect through anxiety alone (PTS → CSA → CST: *β* = 0.066), which may reflect an inconsistent mediation or suppressor-like pattern. This suggests that, after self-efficacy is taken into account, teacher support may in some cases be associated with slightly greater performance-related tension. Several theoretical explanations warrant consideration.

One possible explanation is that supportive teachers may also increase students’ awareness of classroom expectations or participation demands. When teachers are warm and encouraging, students may feel greater obligation to reciprocate by participating, transforming support into subtle performance demand (Jiang and Tanaka, 2022). This aligns with self-determination theory’s distinction between autonomy support versus controlling support: support perceived as controlling can undermine intrinsic motivation and increase pressure.

Second, cultural factors may play a role. In Chinese educational contexts characterized by high power distance, even well-intentioned teacher attention can be interpreted as pressure to perform or avoid disappointing authority figures (Tulis, 2013). Students who perceive high levels of support may also perceive stronger expectations to participate, which could increase anxiety when they remain uncertain about their speaking ability.

However, this positive indirect effect was small relative to the overall negative indirect association between teacher support and silence. Taken together, the results indicate that the confidence-building pathways were more influential than the anxiety-elevating pathway. Practically, this implies educators should focus on competence-oriented support rather than purely affective support to minimize unintended pressure while maximizing empowerment.

### 4.4 Practical implications for vocational education

Based on the results, several strategies are suggested for reducing the cycle of silence in vocational classrooms. First, teacher training programs in vocational education should broaden the notion of support beyond being friendly or encouraging. Teachers need concrete techniques to help students build communicative skills and confidence. Implementing scaffolding strategies such as Think-Pair-Share can ease students into speaking by moving from individual thinking, to peer discussion, and then to whole-class sharing. Sentence starters or response templates may also help less confident students formulate answers.

Second, schools and instructors should intentionally incorporate low-stakes speaking opportunities into everyday classroom activities. Short, ungraded presentations, brief oral summaries, or small-group reporting exercises can serve as manageable mastery experiences. The idea is akin to systematic desensitization in psychology: start with very low-pressure speaking exercises to give anxious students early successes, then progressively raise challenge (Tao et al., 2022).

Third, educators should explicitly address fear of making mistakes. This can be done by establishing classroom norm that mistakes and misunderstandings are natural part of learning (applying Dweck’s growth mindset principle to classroom interaction) (Justice et al., 2023). Teachers might share stories of how others overcame speaking anxiety or how practice leads to improvement. Given that many vocational students feel labeled as “academic failures,” it is crucial to break this learned helplessness.

Fourth, while building efficacy remains the priority, students benefit from coping tools they can use in the moment. Simple techniques like brief deep-breathing exercise before presentation or cognitive reframing strategies from CBT can be taught (Botes et al., 2020). For example, student who feels heart race when about to speak can be taught to take slow breath and remind themselves, “It’s just excitement; I’ve prepared and I can do this.”

### 4.5 Limitations and future directions

While this study provides novel insights into mechanisms of classroom silence, several limitations warrant acknowledgment. First, the cross-sectional design limits the ability to draw firm causal conclusions. The proposed ordering of teacher support, self-efficacy, anxiety, and silence is grounded in Social Cognitive Theory and Control-Value Theory, but the data cannot establish temporal precedence. Some associations may be reciprocal or bidirectional. For instance, more vocal classes may elicit more supportive behavior from teachers, and students with lower anxiety may perceive their teachers more positively. Longitudinal designs following students over time, cross-lagged models, or intervention studies would be needed to test causal direction more rigorously.

Second, focus was on defensive silence as general tendency, but not all silence is necessarily problematic. Some students might be silent for strategic or reflective reasons (what might be called positive or neutral silence). The CST scale aimed to capture negative, withdrawal-type silence, but it would be useful for future research to disentangle different qualities of silence. Qualitative methods or observational studies could help identify when silence signals engagement (e.g., listening intently) versus disengagement (tuning out or freezing up from anxiety) (Botes et al., 2020).

Third, context was limited to vocational universities in a single province of China. Caution is needed in generalizing to other educational settings. Vocational undergraduates have unique characteristics: many are first-generation college students and carry self-concepts shaped by prior academic disappointments. Effects observed might differ in more academically elite universities or in cultural contexts where classroom communication norms are different. Future studies could test model in different countries or at different education levels to examine its robustness (Chen et al., 2025).

Finally, data relied solely on self-report questionnaires. These were appropriate for internal states like self-efficacy and anxiety, but there is room to enrich evidence with more objective measures. Physiological measures (such as heart rate or cortisol levels during class presentations) could provide insight into anxiety experience beyond self-reports. Behavioral observations (like coding frequency and duration of each student’s speaking turns in class, possibly through video or audio recordings) would help validate whether reductions in self-reported anxiety and increases in efficacy actually manifest in more speaking behavior (Zhang et al., 2022).

## 5 Conclusion

Vocational students in China are not uncommonly described as passive or disengaged. This characterization is both partially accurate and incomplete. It is accurate in the sense that silence tendency in the sampled classrooms remained above the midpoint across demographic groups. It is incomplete because the findings suggest that silence is associated with identifiable psychological conditions rather than fixed dispositional passivity. Within the limits of a cross-sectional design, the results indicate that these conditions may be meaningfully addressed through support that builds speaking self-efficacy and reduces anxiety.

The central finding qualifies a common pedagogical intuition: perceived teacher support did not show a significant direct statistical pathway to classroom silence once self-efficacy and anxiety were accounted for (*β* = 0.101, *p* = 0.495). Warm and available teachers remain important, but the results suggest that support is most relevant when students convert it into a stronger appraisal of their own speaking capability. When that appraisal is low, even genuinely supportive teaching may leave silence intact. This is not a pessimistic conclusion; rather, it points to the importance of precise, competence-oriented intervention.

The serial mediation chain quantified here situates the primary psychological bottleneck at self-efficacy rather than anxiety alone. Teacher support was positively associated with self-efficacy (*β* = 0.490), self-efficacy was negatively associated with anxiety (*β* = −0.517), and anxiety was positively associated with silence (*β* = 0.501). Programs that address speaking anxiety only through relaxation techniques or mindfulness exercises, without cultivating genuine communicative competence, may be targeting a downstream symptom rather than a central psychological pathway. The findings therefore suggest that teacher training and communication curricula in vocational institutions should sequence confidence-building opportunities before, or alongside, anxiety-management support.

The suppression effect identified in the anxiety-only pathway (*β* = 0.066) warrants careful attention in subsequent research. The positive residual association between teacher support and anxiety, after self-efficacy variance is partialled out, aligns with performance-pressure dynamics characteristic of high power-distance educational environments. Whether this reflects heightened expectation load, increased classroom visibility, or something more specific to the stigmatized standing of vocational students remains an open empirical question. The current data can identify the pattern with precision; explaining its mechanism will require qualitative follow-up and longitudinal designs that the present study was not structured to provide.

Certain limitations bear directly on the scope of these conclusions. The cross-sectional design limits causal inference, and restriction to universities within a single province raises questions about generalizability. Whether the mechanism holds in vocational institutions elsewhere in China, or in systems outside China where the social meaning of vocational education differs substantially, is empirically open. The exclusive reliance on self-report measures also means that participants’ described silence tendency may not map perfectly onto observable speaking behavior, a gap that future observational or physiological work would be well positioned to close.

Within its scope, however, the study suggests that classroom silence in Chinese vocational higher education is psychologically structured and meaningfully associated with modifiable conditions. The structural model accounted for 52.1% of variance in silence tendency. That figure leaves room for factors not captured here, including peer dynamics, physical classroom design, and instructional format, but it also confirms that the pathway from teacher support through self-efficacy and anxiety carries substantial explanatory weight. For practitioners working in vocational settings where student voice is routinely undersupplied, that pathway is a concrete and evidence-grounded place to begin.

## Data Availability

The datasets presented in this study can be found in online repositories. The names of the repository/repositories and accession number(s) can be found at: https://doi.org/10.6084/m9.figshare.31429667.
